# Evidence for the differential expression of a variant EGF receptor protein in human prostate cancer

**DOI:** 10.1054/bjoc.1999.0898

**Published:** 1999-12-08

**Authors:** E O Olapade-Olaopa, D K Moscatello, E H MacKay, T Horsburgh, D P S Sandhu, T R Terry, A J Wong, F K Habib

**Affiliations:** Departments of Urology and ^3^Pathology, Pathology, Leicester General Hospital, Gwendolen Road, Leicester, LE5 4PW, UK; Kimmel Cancer Institute, 1002 BLSB, 233 S 10th Street, Philadelphia, PA, 19107, USA; University Department of Surgery, Western General Hospital, Crewe Road, Edinburgh, EH4 2XU, UK

**Keywords:** tumour marker, stromal–epithelial interaction, EGFR, prostate/prognostic factors, hormone resistance

## Abstract

Earlier studies have demonstrated an unexplained depletion of the epidermal growth factor receptor (EGFR) protein expression in prostatic cancer. We now attribute this phenomenon to the presence of a variant EGFR (EGFRvIII) that is highly expressed in malignant prostatic neoplasms. In a retrospective study, normal, benign hyperplastic and malignant prostatic tissues were examined at the mRNA and protein levels for the presence of this mutant receptor. The results demonstrated that whilst EGFRvIII was not present in normal prostatic glands, the level of expression of this variant protein increased progressively with the gradual transformation of the tissues to the malignant phenotype. The selective association of high EGFRvIII levels with the cancer phenotype underlines the role that this mutant receptor may maintain in the initiation and progression of malignant prostatic growth, and opens the way for new approaches in the management of this disease including gene therapy. © 2000 Cancer Research Campaign


*British Journal of Cancer* (2000) **82**(1), 186–194
© 2000 Cancer Research Campaign
Article no. bjoc.1999.0898

### Evidence for the differential expression of a variant EGF receptor protein in human prostate cancer

**EO Olapade-Olaopa[1], DK Moscatello[2], EH MacKay[3], T Horsburgh[1], DPS Sandhu[1], TR Terry[1], AJ Wong[2] and FK Habib[4]**

Departments of [1]Urology and [3]Pathology, Leicester General Hospital, Gwendolen Road, Leicester LE5 4PW, UK; [2]Kimmel Cancer Institute, 1002 BLSB, 233 S 10th Street, Philadelphia, PA 19107, USA; [4]University Department of Surgery, Western General Hospital, Crewe Road, Edinburgh EH4 2XU, UK.

**Summary** Earlier studies have demonstrated an unexplained depletion of the epidermal growth factor receptor (EGFR) protein expression in prostatic cancer. We now attribute this phenomenon to the presence of a variant EGFR (EGFRvIII) that is highly expressed in malignant prostatic neoplasms. In a retrospective study, normal, benign hyperplastic and malignant prostatic tissues were examined at the mRNA and protein levels for the presence of this mutant receptor. The results demonstrated that whilst EGFRvIII was not present in normal prostatic glands, the level of expression of this variant protein increased progressively with the gradual transformation of the tissues to the malignant phenotype. The selective association of high EGFRvIII levels with the cancer phenotype underlines the role that this mutant receptor may maintain in the initiation and progression of malignant prostatic growth, and opens the way for new approaches in the management of this disease including gene therapy. © 2000 Cancer Research Campaign

**Keywords:** tumour marker; stromal–epithelial interaction; EGFR; prostate/prognostic factors; hormone resistance

Signalling between polypeptide growth factors and their specific receptors is an integral part of the molecular pathways that regulate the normal and abnormal growth of solid organs (Cross and Dexter, 1991). The epidermal growth factor receptor (EGFR) is a 170 kDa transmembrane receptor that plays an important role in the differentiation and proliferation of epithelial cells (Thompson and Gill, 1985). Binding of the receptor by any of its ligands (e.g. the epidermal growth factor [EGF], transforming growth factor-alpha [TGF-α] or amphiregulin [AR]) results in its activation and the initiation of a cascade of reactions that ultimately result in DNA replication and cell division (Gullick, 1998). The EGFR has previously been implicated in the malignant transformation of epithelial cells, and high levels of EGFR mRNA and protein have been found in several solid organ malignancies (Ozanne et al, 1986; Sainsbury et al, 1987; Derynck et al, 1993; Barlett et al, 1996). Amplification and rearrangement of the EGFR gene has also been associated with over-expression of the receptor protein in several solid tumours (Xu et al, 1984; Hunts et al, 1985; RO et al, 1988; Wong et al, 1990).

Earlier reports on EGFR expression in prostatic tumours are conflicting. Whilst the expression of EGFR mRNA is increased in these neoplasms (Davies et al, 1988; Morris and Dodd, 1990; Tukeri et al, 1994; Glynne-Jones et al, 1996), some studies have shown a gradual decrease in the expression of the receptor protein with increasing malignant transformation of the epithelial cells (Maddy et al, 1989; Mellon et al, 1992; Tukeri et al, 1994). However, other studies reported similar levels of EGFR protein expression in benign prostatic hyperplasia (BPH) and prostate cancer (CaP) (Cohen et al, 1994; Glynne-Jones et al, 1996). We postulated that these contradictory findings were due to the expression of a mutated EGFR by prostatic tumours.

Several mutations of EGFR have been found in tumours (Ekstrand et al, 1992; Moscatello et al, 1995; Panneerselvam et al, 1995). The most common of these variant receptors is the EGFRvIII which has been detected in several cancers (Moscatello et al, 1995). EGFRvIII results from the deletion of exons 2–7, which leads to an 801-bp in-frame deletion of the external domain of the normal receptor (Wong et al, 1990). This rearrangement removes most of the first two extracellular sub-domains of EGFR, but preserves the reading frame of the receptor message. As such, this aberrant EGFR does not bind any known EGFR ligand, but is a constitutively active tyrosine kinase which initiates mitosis independent of ligand-binding. The EGFRvIII has been detected in tumour cells only and is thought to play a significant role in the pathogenesis and aggressiveness of cancers (Kristt and Yarden, 1996; Moscatello et al, 1996; Nagane et al, 1996).

There are no previous reports of EGFRvIII detection in prostatic tumours. Using an antibody that has previously been shown to be highly specific for this variant receptor (Humphrey et al, 1990; Wikstrand et al, 1995), as opposed to the native EGFR (WT-EGFR), we investigated EGFRvIII expression in both benign and malignant prostatic neoplasms. We have also compared its expression with that of WT-EGFR in these tissues, and our results support our hypothesis that the reported reduction of WT-EGFR in prostatic malignancy is due to the expression of an altered form of the receptor. In addition, we evaluated the clinical significance of EGFRvIII expression in CaP and found that the over-expression of this aberrant EGFR is predictive of an aggressive phenotype of the disease.

*Received 24 March 1999*
*Revised 22 June 1999*
*Accepted 7 July 1999*

*Correspondence to:* EO Olapade-Olaopa, Section of Urology, Department of Surgery, The University of Michigan Medical Center, 1500 E Medical Center Drive, Ann Arbor, MI 48109-0330, USA

This project is dedicated to Professor BO Osuntokun (*beate memoriae*) and Mr PT Doyle (*beate memoriae*).

These results were presented in part at the Schilling Research Conference, Hormones and Cancer in Santa Cruz, CA, 18–21 September 1997, and the International Symposium of the British Prostate Group in York, UK, 2–4 October 1997.

### MATERIALS AND METHODS

### Clinical materials

#### Fresh-frozen material

Fresh-frozen tissue for reverse transcription polymerase chain reaction (RT-PCR) and Western blotting tests were obtained during transurethral resecection of BPH and CaP glands. The chips were flash frozen to –170°C in liquid nitrogen immediately after evacuation from the bladder. Routine haematoxylin and eosin staining was done to confirm histological diagnosis. Chips were then chosen for subsequent analysis if shown to be either wholly infiltrated by malignant cells with little intervening stroma (CaP) or consisting largely of benign hyperplastic glands (BPH).

#### Archival material

Sections prepared from archival surgical specimens obtained in 1993 from 38 patients with CaP (31 newly diagnosed and seven hormone-resistant cases) and 19 age-matched patients with BPH, were supplied by the Pathology Department of Leicester General Hospital. Paraffin sections from 12 archival metastatic deposits (six bone and six lymph node) supplied by the Department of Surgery, Western General Hospital, Edinburgh were also available for screening. Histological evaluation was performed by routine haematoxylin and eosin staining of representative sections from test tissues. The primary CaP specimens were graded using the Gleason score (Gleason et al, 1974), and classified as benign, well differentiated (Gleason score 2–4), moderately differentiated (Gleason score 5–7) or poorly differentiated (Gleason score 8–10). High-grade prostatic intra-epithelial neoplasia (HG PIN) was seen in sections from 14 CaP glands.

**Table 1** Expression of WT-EGFR in prostatic tissues

| Histology (Total no.) | Intensity of immunoreaction | | | |
|---|---|---|---|---|
| | Strong (%) | Moderate (%) | Weak (%) | Absent (%) |
| Normal/atrophic (19) | 17/19 (89) | 2/19 (11) | 0/19 (0) | 0/19 (0) |
| BPH (19) | 13/19 (68) | 6/19 (32) | 0/19 (0) | 0/19 (0) |
| HG PIN (14) | 0/14 (0) | 6/14 (43) | 8/14 (57) | 0/14 (0) |
| CaP (Cum) (38) | 3/38 (8) | 4/38 (11) | 26/38 (68) | 5/38 (13) |
| G1 (11) | 2/11 (18) | 2/11 (18) | 5/11 (46) | 2/11 (18) |
| G2 (10) | 0/10 (0) | 1/10 (10) | 8/10 (80) | 1/10 (10) |
| G3 (17) | 1/17 (6) | 1/17 (6) | 13/17 (76) | 2/17 (12) |
| Metastases (12) | 0/12 (0) | 1/12 (8) | 1/12 (8) | 10/12 (83) |
| Bone (6) | 0/6 (0) | 1/6 (17) | 0/6 (0) | 5/6 (83) |
| Lymph (6) | 0/6 (0) | 0/6 (0) | 1/6 (17) | 6/6 (100) |

BPH, benign prostatic hyperplasia; HG PIN, high-grade prostatic intra-epithelial neoplasia; CaP, carcinoma of the prostate; Cum, cumulative scores. Detailed distribution of WT-EGFR scores in normal/atrophic, benign hyperplastic, partially transformed and primary and metastatic malignant prostatic tissues. Despite the progressive decrease in WT-EGFR expression with increasing de-differentiation of the glandular cells, WT-EGFR scores within each tissue histotype was variable.

### Clinical data

Data were available for retrieval in all 38 CaP patients, and was complete in 34 (90%). All data were included for analyses. The minimum time interval between tissue retrieval and data review was 36 months. Clinical parameters recorded in addition to age and histological grade were: (1) serum PSA, (2) hormone status, (3) presence of metastasis at diagnosis, (4) time to disease progression following hormonal therapy (a rise in PSA level by greater than twice the nadir level, or the appearance of new metastases), (5) survival status, (6) follow-up period or length of survival. The minimum follow-up period was 36 months and the maximum was 52 months, with a median of 37 months (mean 39.2 months). Thirteen patients died before the review, and all but 1 death was from CaP-related causes (as recorded in the death certificates).

#### RT-PCR

RNA was isolated from fresh-frozen BPH and CaP tissue with RNAzol B (Tel-Test, Inc., Friendswood, TX, USA) according to the supplied protocol. Reverse transcription (RT) reactions were done using 4 μg total RNA and Superscript II Reverse Transcriptase from Gibco-BRL according to the manufacturer's protocol. *Taq* Polymerase was from Boehringer Mannheim (Indianapolis, IN, USA), dNTPs were from Pharmacia Biotech (Piscataway, NJ, USA), and the primers were synthesized in the Nucleic Acid Core Facility in the Kimmel Cancer Institute (KCI). The following primers were used (nucleotides corresponding to the published WT-EGF receptor cDNA (Ulrich et al, 1984)): 5′ primers: (1) nt142–159: 5′ CAg TAT TgA TCg ggA gAg 3′; (2) nt180–197: 5′ AgC AgC gAT gCg ACC CTC 3′; (3) nt250–266: 5′ AgT Cgg gCT CTg gAg gA 3′; 3′ primers: (4) nt1285–1268: 5′ CAC TgA Tgg Agg TgC AgT 3′; and (5) nt1140–1123: 5′ CAT CTC ATA gCT gTC ggC 3′. Primer 4 was used in the RT reaction, the first PCR reaction used primers 1 and 4, the second PCR reaction used primers 2 and 4, and the third reaction used primers 3 and 5. PCR conditions were as previously described (Wong et al, 1992), except that the annealing temperature used was 53°C. PCR products were separated on 1.5% agarose TAE gels at 80 V for 1–1.5 h. PCR products were purified for secondary PCR reactions using the QiaQuick PCR Purification Kit, and for sequencing using the Gel Extraction Kit, both from Qiagen (Santa Clarita, CA, USA). PCR products were sequenced using PRISM Ready Reaction DyeDeoxy Terminator Cycle Sequencing on an Applied Biosystems Model 377 at the KCI Nucleic Acid Facility.

#### Antibodies

The antibodies used in this study were: (1) a commercially available mouse monoclonal anti-EGFR (clone 31G7, Zymed, San Francisco, CA, USA) which reacts with the peptide backbone of EGFR and recognizes the WT-EGFR only (Sainsbury et al, 1987); and (2) a rabbit polyclonal antibody to EGFRvIII produced by DKM and AJW. This antibody was raised against pepEGFRvIII (LEEKKGNYVVTDHC) and affinity-purified as previously described (Humphrey et al, 1990).

#### Western blotting

Lysates were prepared by homogenizing fresh-frozen tissue samples of BPH and CaP shown by immunohistochemistry (see below) to express both forms of EGFR in buffer. Western blot analysis was carried out as previously described (Laemmli, 1970). Briefly, 250 μg of protein from BPH and CaP samples along with aliquots of control lysates (HC2 20d/2c tumour; EGFRvIII positive control, A431 tumour; WT-EGFR positive control, Jurkat human T-cell lymphoma; WT-EGFR and EGFRvIII negative control), were separated on 6% sodium dodecyl sulphate (SDS)-polyacrylamide gels and transferred onto nitro-cellulose membranes (Amersham, Bucks, UK). The membranes were incubated with either polyclonal

© 2000 Cancer Research Campaign

**Table 2** Expression of EGFRvIII in prostatic tissues

| Histology (Total no.) | Intensity of immunoreaction | | | |
|---|---|---|---|---|
| | Strong (%) | Moderate (%) | Weak (%) | Absent (%) |
| Normal/Atrophic (19) | 0/19 (0) | 0/19 (0) | 0/19 (0) | 0/19 (0) |
| BPH (19) | 0/19 (0) | 2/19 (11) | 17/19 (89) | 0/19 (0) |
| HG PIN (14) | 4/14 (29) | 10/14 (71) | 0/14 (0) | 0/14 (0) |
| CaP Cum (38) | 26/38 (68) | 10/38 (26) | 2/38 (5) | 0/38 (0) |
| G1 (11) | 4/11 (36) | 5/11 (46) | 2/11 (18) | 0/11 (0) |
| G2 (10) | 7/10 (70) | 3/10 (30) | 0/10 (0) | 0/10 (0) |
| G3 (17) | 15/17 (88) | 2/17 (12) | 0/17 (0) | 0/17 (0) |
| Metastases (12) | 11/12 (92) | 1/12 (8) | 0/12 (0) | 0/12 (0) |
| Bone (6) | 6/6 (100) | 0/6 (0) | 0/6 (0) | 0/6 (0) |
| Lymph (6) | 5/6 (83) | 1/6 (17) | 0/6 (0) | 0/6 (0) |

BPH, benign prostatic hyperplasia; HG PIN, high-grade prostatic intra-epithelial neoplasia; CaP, carcinoma of the prostate; Cum, cumulative scores. Detailed distribution of EGFRvIII scores in normal/atrophic, benign hyperplastic, partially transformed and primary and metastatic malignant prostatic tissues. As seen in anti-WT-EGFR immunostaining there was variability in the level of EGFRvIII expression within individual histological grades.

anti-EGFRvIII rabbit antibody, or anti-EGFR mouse monoclonal antibody. Secondary biotinylated antibodies (Dako, Cambs, UK) were applied to the membranes which were then developed according to Amersham's enhanced chemiluminescence (ECL) protocol.

### *Immunohistochemistry*

Anti-WT-EGFR and anti-EGFRvIII staining was detected by streptavidin–biotin amplified immunoperoxidase reactivity. Serial sections from primary and metastatic prostatic tissues were stained within 3 weeks of preparation to exclude antigen degradation as previously described (Olapade-Olaopa et al, 1998). Briefly, after preparation and exposure of the antigenic epitopes (microwave and pronase treatment for EGFRvIII and WT-EGFR respectively), the sections were incubated with the relevant primary (anti-EGFRvIII or anti-EGFR) and secondary antibodies using the Dako detection kit. Positive and negative controls were included in all staining runs. The positive controls were: (1) normal skin for WT-EGFR; (2) HC2 20d/2c mouse tumour (EGFRvIII). Negative controls were: (1) monoclonal anti-mouse IgG (WT-EGFR); (2) normal rabbit serum (EGFRvIII); and (3) normal goat serum. Antibody cross-reactivity was excluded by staining WT-EGFR and EGFRvIII positive CaP sections pre-incubated with recombinant EGF (blocking 31G7 binding but not EGFRvIII) with both antibodies, and by incubating sections of HC2 20d/2c mouse tumour with anti-WT-EGFR. These sections were negative for WT-EGFR but positive for EGFRvIII.

### Evaluation of immunochemistry

Immuno-reactivity of homogeneous histological areas within the sections was assessed independently, and without prior knowledge of histological grading by an experienced pathologist and scored using a modification of the H-scoring system (Newby et al, 1997). Briefly, the intensity of the reaction (0–3+) in homogeneous histological areas of the sections was weighted by the percentage of cells staining at each intensity. Thus each antibody staining had a range from 0 (no staining) to 300 (100% 3+ staining). The final scores were classified as: 0 = negative, 1–33% = low expression, 34–66% = moderate expression, > 66% = high expression.

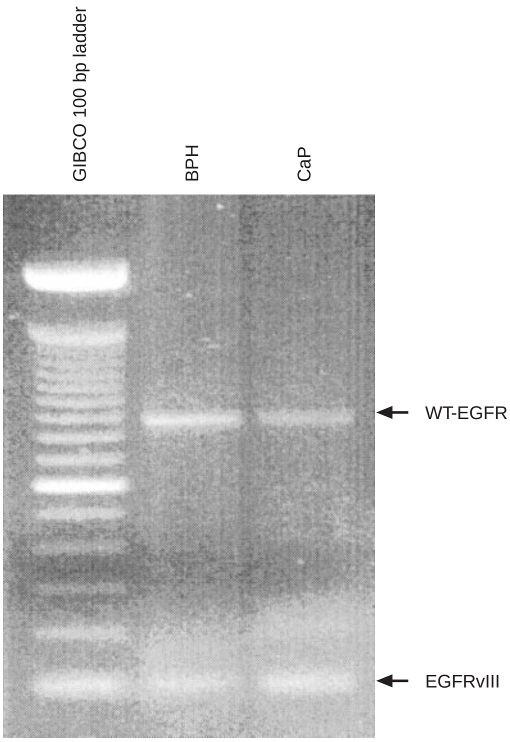

**Figure 1** RT-PCR detection of WT-EGFR and EGFRvIII mRNA transcripts in BPH and CaP. The upper arrow indicates the expected 890 bp WT-EGFR band and the lower arrow the 89 bp EGFRvIII band generated by nested PCR using primers 3 and 5 as described in the Methods. Immunohistochemical staining of representative sections from these specimens showed high WT-EGFR and low EGFRvIII levels in the BPH tissue, whilst the CaP sample stained strongly for EGFRvIII and weakly for WT-EGFR

### Statistical methods

The results were reported as proportions and mean scores (± s.d.). Statistical comparison of mean WT-EGFR and EGFRvIII scores in individual histological groups was done using the paired and unpaired two-sample (adjusting for unequal standard deviations) *t*-tests as appropriate. Spearman's correlation coefficient was used to examine the univariate associations between EGFRvIII expression and the clinical indices. Multivariate analysis of the expression of EGFRvIII and the prognostic parameters was done using Cox's proportional hazards regression. The impact of EGFRvIII over-expression on survival was studied using the Kaplan–Meier method. All tests were two-sided where appropriate and were performed at the two-sided 0.05 level of significance.

### RESULTS

### Identification of wild-type and variant EGFR mRNA in prostatic tumours

Transcripts for the two types of EGFR were detected in both BPH and CaP. However, the WT-EGFR mRNA was more easily amplified from both specimens than the EGFRvIII mRNA, as EtBr-stained

 © 2000 Cancer Research Campaign

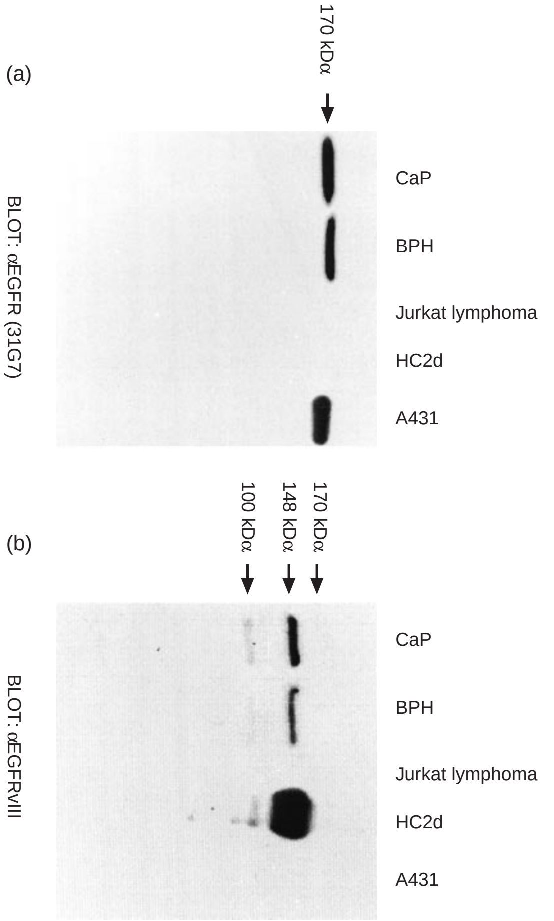

**Figure 2** Expression of WT-EGFR and EGFRvIII in BPH and CaP analysed by Western blotting. Aliquots of lysates containing 250 µg of BPH and CaP, 25 µg of HC2 20d/2c tumour (EGFRvIII positive control), 50 µg of A431 tumour (WT-EGFR-positive control), and 50 µg of Jurkat human T-cell lymphoma (EGFR-negative control) were added to the wells. The lysates were run on 7.5% PAGE at 200 mV, and then immunoblotted with antibodies to either the wild-type or variant EGF receptor. A single band of proteins was recognized in the 170 kDa region in BPH and CaP by anti-WT-EGFR clone 31G7, whilst bands of protein were detected by the anti-EGFRvIII antibody in BPH and CaP lysates in the 140 kDa region. A 90 kDa band was also seen in the HC2 20d/2c and CaP lanes of the EGFRvIII blot confirming the expression of multiple isoforms of the mutated protein by tissues which overexpress the EGFRvIII as previously reported (Ekstrand et al, 1992; Moscatello et al, 1995, 1996). Specificity of the antibodies used is confirmed by the strong anti-WT-EGFR signal seen at 170 kDa region in A431 lane and anti-EGFRvIII signal seen in the HC2 20d/2c lane in the absence of cross-reactions (signals) in these respective lanes in the immunoblots for each antigen. There were no signals in the negative control lanes in either blot

WT-WGFR bands could be seen after nested PCR, but a tertiary PCR reaction was necessary to visualize the EGFRvIII band (Figure 1 and data not shown).

### Detection of WT-EGFR and EGFRvIII proteins and confirmation of antibody specificity

Anti-EGFRvIII immunoblotting detected a 148-kDa band in BPH and CaP lysates which corresponds to the variant EGFR (Figure 2A). A weak EGFRvIII signal was also seen in the 90 kDa region in the CaP lysate. On the other hand, Western blot analysis of these samples using the anti-WT-EGFR detected a 170 kDa protein only, corresponding to the native EGFR in both tumour lysates (Figure 2B). As such, as well as detecting the presence of both WT-EGFR and EGFRvIII protein, these results further confirmed the specificity of the antibodies for their respective antigens.

### Localization of WT-EGFR and EGFRvIII immunostaining in prostatic tissues

Primary prostatic tumours (BPH and CaP) are heterogeneous diseases, and glands of different histotypes may be adjacent to each other within tumour sections. We found that WT-EGFR and EGFRvIII pattern of staining was dependent on the histology of the individual glands within our sections as similar immuno-reactions were seen in normal/atrophic and BPH glands in both BPH and CaP sections. We also determined that WT-EGFR expression was mainly membranous and that staining was strongest in the basal cells of the glands (Figure 3 A–D). In contrast, although some membranous EGFRvIII staining was seen, the variant antigen was expressed mainly in the cytoplasm, and this for the most part appeared as a distinct peri-nuclear deposit on the luminal surface of the cells (Figure 4 B–D).

### WT-EGFR protein expression is decreased in prostatic neoplasms

The highest expression of WT-EGFR protein was seen in normal/atrophic glands, and the mean expression of this normal receptor decreased as the epithelial cells de-differentiated (Table 1A and Figure 3A–G). Statistical comparison of the mean scores in the various histological groups revealed that the progressive decrease in WT-EGFR was highly significant, i.e. normal/atrophic vs BPH, and BPH vs HG PIN and CaP glands ($P \leq 0.0001$). WT-EGFR expression was also significantly lower in metastatic deposits than in CaP glands ($P = 0.015$). However, the levels of expression of the normal receptor in HG PIN and CaP glands were statistically similar ($P = 0.41$), as were the mean scores in early and advanced CaP tumours (G1 vs G2/3) ($P = 0.12$).

### EGFRvIII protein is expressed by neoplastic prostatic cells only

In contrast to the normal receptor, EGFRvIII was expressed by abnormal prostatic epithelial cells only and not by normal glands. Furthermore, the mean EGFRvIII expression increased as the tumours became more malignant with poorly differentiated tumours and metastases staining the strongest (Table 1B and Figure 4A–G). Statistical comparison of the scores revealed significant differences between EGFRvIII expression in benign and partially or fully transformed glands: BPH vs HG PIN or CaP ($P = < 0.0001$), and also between CaP and metastases ($P = 0.004$). However, in contrast to WT-EGFR, EGFRvIII expression in the higher grade tumours (G2 and G3) was significantly higher than in well-differentiated (G1) tumours ($P = 0.006$).

### Hormone-resistant CaP glands have higher levels of EGFRvIII protein expression

We compared EGFRvIII and WT-EGFR expression in newly diagnosed (hormone-naive) and hormone-resistant glands. We found that whilst EGFRvIII expression was significantly higher in hormone resistant CaP than in untreated glands ($P = 0.012$), mean WT-EGFR expression was similar in the two groups ($P = 0.86$).

*© 2000 Cancer Research Campaign* 

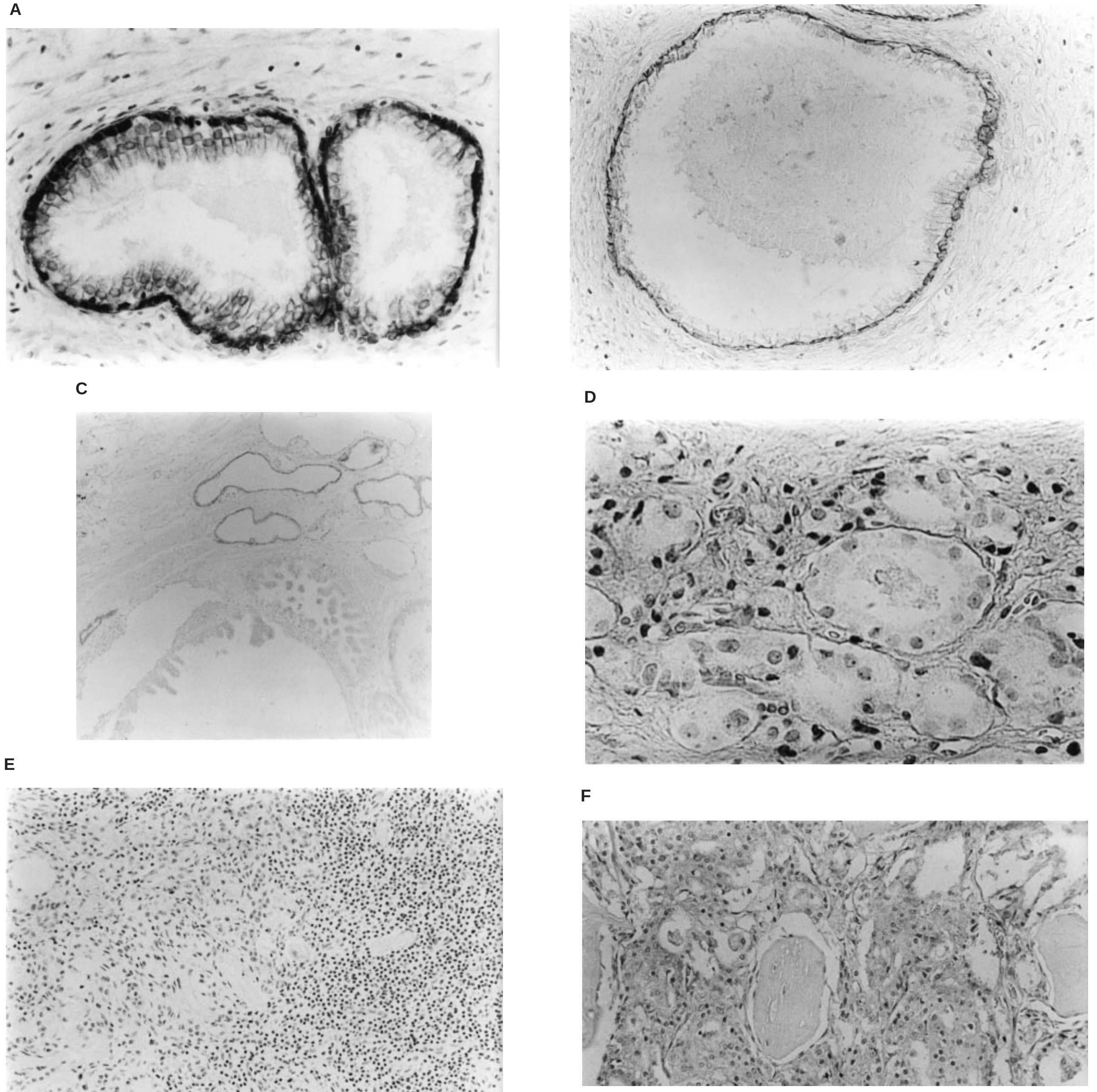

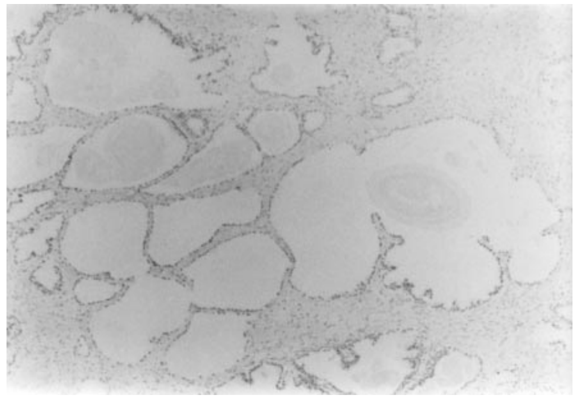

**Figure 3** WT-EGFR immunostaining in prostatic tumours (× 250). Sections were counterstained with haematoxylin. (**A**) Normal/atrophic glands showing high WT-EGFR expression. (**B**) BPH glands showing moderate expression of WT-EGFR. (**C**) HG PIN glands showing minimal WT-EGFR staining of transformed cells whilst basal cells of adjacent benign gland showed strong immunoreactivity. (**D**) Weak WT-EGFR expression in CaP glands. (**A–D**) Membranous WT-EGFR immunoreaction was strongest in basal cells and appeared as an outer rim surrounding the glands. (**E**) Lymph node metastasis showing no WT-EGFR staining in cells of prostatic or lymphoid origin. (**F**) Metastatic prostatic deposit in bone also showing no WT-EGFR expression in either the invading or native cells of the tissue. (**G**) WT-EGFR negative control (BPH section × 100)

*© 2000 Cancer Research Campaign*

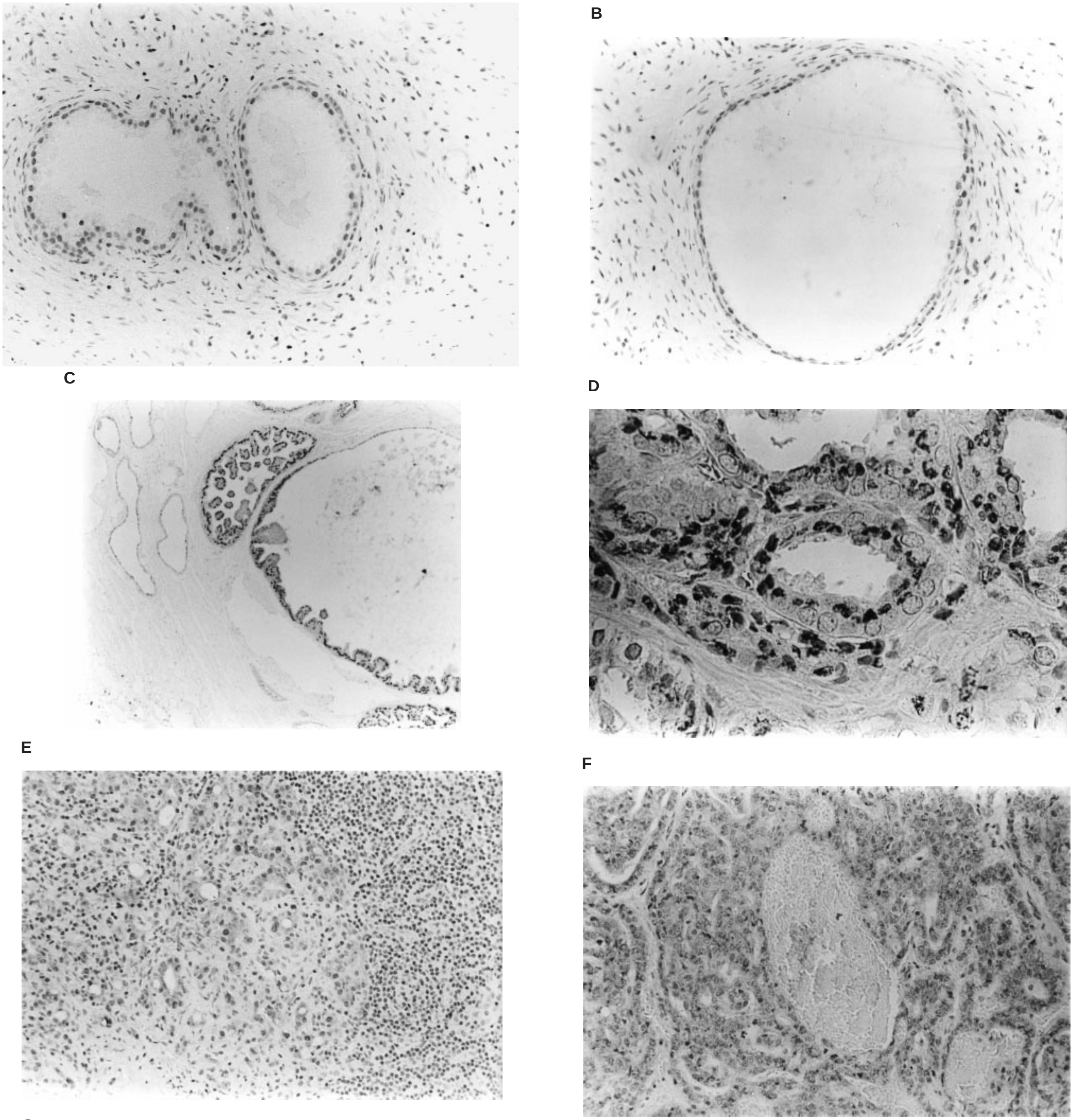

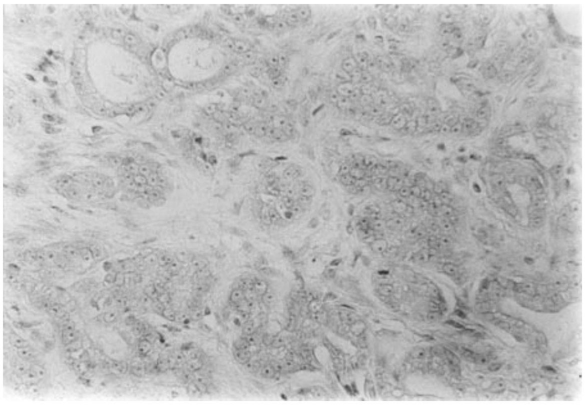

**Figure 4** EGFRvIII immunostaining in serial sections from prostatic tumours counterstained with haematoxylin (×250). (**A**) EGFRvIII was not expressed in normal/atrophic glands. (**B**) Weak EGFRvIII immuno-reaction in BPH glands. (**C**) HG PIN gland showing strong EGFRvIII staining in fully transformed cells whilst histologically benign cells within the gland and the adjacent BPH gland stained weakly. (**D**) Strong expression of EGFRvIII in CaP glands. (**A–D**) Cytoplamic EGFRvIII staining was seen mainly as a perinuclear deposit on the luminal side of the tumour cells and gave an impression of an inner rim within fully formed glands. (**E**) Metastatic prostatic cells in a lymph node showing strong EGFRvIII staining in the midst of negative lymphoid cells. (**F**) CaP deposit in bone showing high EGFRvIII expression in the metastatic cells whilst surrounding osteocytes are negative (**G**) EGFRvIII-negative control (CaP section × 100)

*© 2000 Cancer Research Campaign*

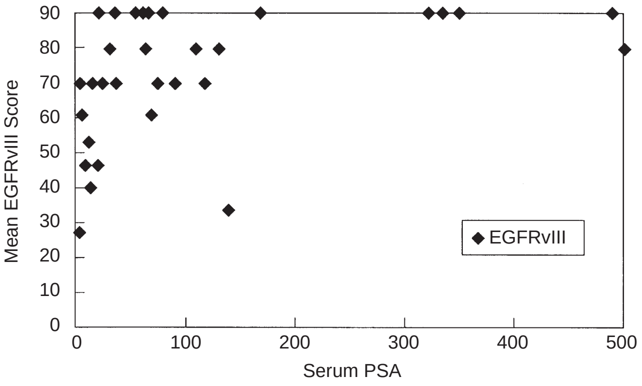

**Figure 5** A scatter graph comparing EGFRvIII scores with serum PSA levels in individual CaP patients. This showed a significant association between EGFRvIII expression and serum PSA level at the time of tissue retrieval ($P = 0.005$)

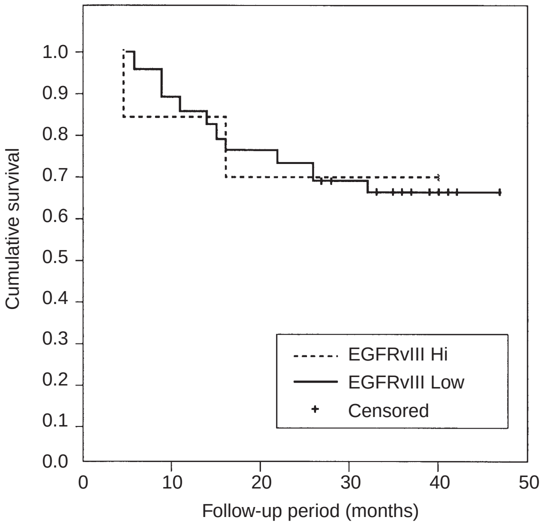

**Figure 6** Kaplan–Meier overall survival estimates showing minimal effect of EGFRvIII over-expression on the survival of CaP patients in this study

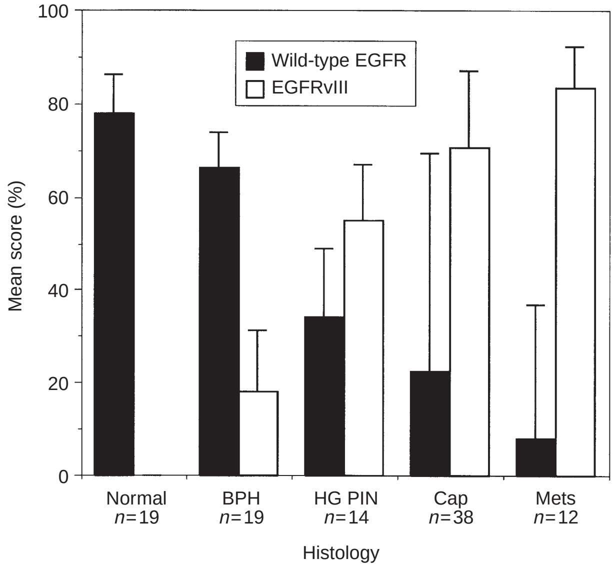

**Figure 7** Graphical representation of WT-EGFR and EGFRvIII mean scores (± s.d.) in the different histological grades of prostatic tissues demonstrating an inverse relationship between the levels of expression of the two types of EGF receptors. The different between WT-EGFR and EGFRvIII mean scores (± s.d.) was statistically significant in each tissue histiotype ($P = < 0.0001$)

Also, co-expression of WT-EGFR and EGFRvIII was seen in 7/7 (100%, confidence interval (CI) 59–100%) of sections from hormone resistant glands as compared to 26/31 (84%, CI 66–95%) of sections from untreated CaP, but this difference was not statistically significant ($\chi^2$, $P = 0.77$).

### EGFRvIII protein expression is indicative of an aggressive prostate cancer phenotype

We assessed the effect of EGFRvIII expression on the clinical course of CaP by correlating EGFRvIII scores with accepted prognostic parameters for survival of patients with CaP (age, serum prostate-specific antigen (PSA), histological grade, and pathological and clinical stage) (Sakr and Grigon, 1997). The duration of response to hormonal manipulation (time to disease progression) is also a useful indicator of the aggressiveness of CaP. EGFRvIII expression was significantly related to serum PSA ($P = 0.005$) (Figure 5), and the time to disease progression ($P = 0.05$) only. At the time of data review, 12 patients had died from CaP-related causes but the level of EGFRvIII expression did not have a significant influence on survival during the follow-up period ($P = 0.83$) (Figure 6).

Univariate analysis of the relationship between the prognostic factors and the survival of our patients revealed that only the time to disease progression following hormone therapy ($P = 0.007$), and the presence of metastasis at diagnosis (i.e. advanced disease at presentation) ($P = 0.04$) had a significant impact on survival. Although EGFRvIII activity was not significant as an individual prognostic factor for CaP, its association with the time to disease progression indicates its over-expression may be predictive of a poor response to hormonal manipulation. Multivariate analysis using Cox's proportional hazards showed that only an advanced clinical stage had a significant influence on survival at 3 years ($P = 0.01$).

### DISCUSSION

Since we first detected the presence of EGFR in BPH (Maddy et al, 1987), there have been conflicting reports on the levels of the receptor protein found in prostatic tumours (Maddy et al, 1989; Maygarden et al, 1992; Mellon et al, 1992; Cohen et al, 1994; Tukeri et al, 1994; Glynne-Jones et al, 1996). In this current study, using two independent investigative techniques, we have shown for the first time the presence of a variant EGF receptor in prostatic tumours. Furthermore, although EGFRvIII has been detected in other cancers, to our knowledge this is the first study to evaluate its association with WT-EGFR in human neoplasms. Despite the small numbers of specimens incorporated in our project, the consistency of the inverse relationship between the level of immuno-reactivity of these two antigens through all stages of prostatic differentiation scrutinized, supports our hypothesis that the progressive decrease in EGFR expression in prostatic neoplasms is due to the differential expression of an altered receptor by abnormal epithelial cells of this gland. As there is

© 2000 Cancer Research Campaign increasing evidence from clinical and basic science research that PIN is a precursor of invasive prostatic disease (Haggman et al, 1997), our findings are further validated by our demonstration that WT-EGFR and EGFRvIII levels in HG PIN were intermediate between those recorded in BPH and CaP glands.

Interestingly whilst both WT-EGFR and EGFRvIII mRNA were detected in both BPH and CaP, the expression of the variant receptor protein increased with de-differentiation of prostatic epithelial cells with a concomitant decrease in WT-EGFR expression (Figure 7). This suggests that as prostatic tumours advance they increasingly express this constitutively active variant protein in preference to the normal receptor. Further support is offered by our finding of a higher expression of EGFRvIII in sections from hormone resistant CaP sections and by previous reports of the detection constitutive EGFR activity in androgen-independent prostate cancer cell lines (Sherwood et al, 1998). We have recently reported that malignant transformation of prostatic epithelium is associated with a loss of androgen receptor expression in stromal cells, and postulated that this may result in the loss of stromal derived epithelial mitogens (ligands) (Olapade-Olaopa et al, 1999). In this event, the reliance of neoplastic prostatic glands on ligand-independent pathways (such as the expression of a constitutively active receptor) may be a response to the cessation of proliferative signals from the stromal compartment.

Although the specificity of our antibodies was confirmed by Western blotting and the inclusion of positive and negative controls in the immuno-staining tests, our immunohistochemical data also showed that the staining patterns of anti-WT-EGFR and anti-EGFRvIII in prostatic tissues differed. Immunohistochemistry affords direct visualization of the cellular component where the antigen–antibody reaction occurs, and we found that whilst WT-EGFR immuno-reactions were on the cell-membrane, EGFRvIII expression was mainly cytoplasmic/perinuclear. The presence of a perinuclear deposit in EGFRvIII-positive prostate cells is similar to earlier observations in glial tumours (Nishakawa et al, 1994; Moscatello et al, 1996), and may be due to the immuno-localization of the internalized variant receptor (following ligand-independent dimerization). The significance of this difference in the cellular distribution of the two types of EGFR is at present unclear.

In common with other studies, this variant EGFR was expressed by prostatic tumour cells only with no evidence for its expression in normal cells. The demonstrable specificity and sensitivity of high EGFRvIII expression by (partially or fully) malignant cells only in HG PIN, CaP and metastases (including those cells that did not stain for PSA), indicates the potential usefulness of the EGFRvIII as a marker when screening tissues for malignant prostatic cells especially when PSA staining is weak or absent (Bostwick et al, 1998).

In previous studies of EGFRvIII expression in glioblastomas, high levels were found preferentially in advanced tumours (grades III and IV) (Wong et al, 1987). Other studies have also shown that EGFRvIII increases the tumorigenicity of malignant epithelial cells by increasing mitosis and reducing apoptosis (Nagane et al, 1996), and also by increasing their metastatic potential (Nishakawa et al, 1994; Moscatello et al, 1996). The increasing levels of EGFRvIII detected as prostatic neoplasms progressed from intraepithelial changes to metastatic disease, and the finding that EGFRvIII expression is predictive of a poor response to hormone therapy, suggests that over-expression of this antigen may contribute to both the malignant transformation of prostatic cells and the subsequent progression to hormone insensitivity. In addition, co-expression of WT-EGFR and EGFRvIII was found in all hormone resistant glands. EGFRvIII is known to increase the mitotic activity of WT-EGFR (Nishakawa et al, 1994), and it is possible that the possession of two different mitotic pathways, which act in synergy, is desirable for hormone-independent proliferation by prostatic cells in vivo.

Growth factors and receptors are recognized as potentially effective targets of anticancer therapies (Greig et al, 1988; Modjtahedi et al, 1996), but the down-regulation of the EGFR protein in prostatic malignancies has limited trials of these treatments in these tumours. However, there are recent reports of promising results with monoclonal antibodies that inhibit constutive EGFR phosphorylation (Fong et al, 1992), as well as drugs directed at the receptor signalling pathways (Putz et al, 1999) in CaP cell lines. We have recently reported that the immunization of immunocompetent laboratory animals with anti-EGFRvIII vaccines stimulated the regression of established tumours (Moscatello et al, 1997). Furthermore, expression of EGFRvIII has been shown to markedly increase the sensitivity of epithelial cells to anti-EGF-receptor-specific toxins (Schmidt et al, 1998). In the light of these reports, the specificity of high EGFRvIII expression for malignant prostatic cells suggests that this variant receptor may be a suitable target for novel treatment options in CaP including gene therapy.

### ACKNOWLEGEMENTS

We thank Professors PRF Bell and M Nicholson, as well as Messrs JT Flynn and RC Kockelbergh, Ms A Lycett and Dr JL Ware (Medical College of Virginia Campus) for their assistance with this project. Also, Mr N Taub for his assistance with the statistical analysis and Mr T Alonge for his comments on the manuscript.

### REFERENCES

Barlett J, Langdon S, Simpson B, Stewart M, Katsaros D, Simondi P, Love S, Scott W, Williams A, Lessells A, Macleod K, Smyth J and Miller W (1996) The prognostic value of epidermal growth factor receptor mRNA expression in primary ovarian cancer. *Br J Cancer* **73**: 301–306

Bostwick D, Pacelli A, Blute M, Roche P and Murphy G (1998) Prostate specific membrane antigen expression in prostatic intraepithelial neoplasia and adenocarcinoma: a study of 184 cases. *Cancer* **82**: 2256–2261

Cohen D, Simark R, Fair W, Melamed J, Scher H and Cordon-Cardo C (1994) Expression of transforming growth factor-alpha and the epidermal growth factor receptor in human prostatic tissue. *J Urol* **152**: 2120–2124

Cross M and Dexter T (1991) Growth factors in development, transformation and tumorigenesis (review). *Cell* **64**: 271–280

Davies P, Eaton C, France T and Philips E (1988) Growth factor receptors and oncogene expression in prostatic cell. *Am J Clin Oncol* **Supplement 2**: S1–S7

Derynck R, Goeddel D, Ulrich A, Gutterman J, Williams R, Bringman T and Berger W (1993) Synthesis of messenger RNAs for transforming growth factors alpha and beta and the epidermal growth receptor by human tumours. *Cancer Res* **47**: 707–712

Ekstrand A, Sugawa N, James C and Collins V (1992) Amplified and rearranged epidermal growth factor genes in human glioblastomas reveal deletion sequences encoding portions of the N- and/or C-terminal tails. *Proc Natl Acad Sci USA* **89**: 4309–4313

Fong C, Sherwood E, Mendelsohn J, Lee C and Kozlowski J (1992) Epidermal growth facor receptor monoclonal antibody inhibits constitutive receptor phosphorylation, reduces autonomous growth, and sensitizes androgen-independent prostatic carcinoma cells to tumour necrosis factor alpha. *Cancer Res* **52**: 5887–5892

Gleason D, Mellinger G and Group, TVACUR (1974) Prediction of prognosis for prostate adenocarcinoma by combined histological grading and clinical staging. *J Urol* **111**: 58–64

© 2000 Cancer Research Campaign

Glynne-Jones E, Goddard L and Harper M (1996) Comparative analysis of mRNA and protein expression for EGFR and ligands: relationship to proliferative index of human prostate tissue. *Hum Pathol* **27**: 688–696

Greig R, Dunnington D, Murthy U and Mario A (1988) Growth factors as novel therapeutic targets in neoplastic disease. *Cancer Surv* **7**: 644–674

Gullick W (1998) Type 1 growth factor receptors: current status and future work. *Biochem Soc Symp* **63**: 193–198

Haggman M, Macoska J, Wonjo K and Oesterling J (1997) The relationship between prostatic intraepithelial neoplasia and prostate cancer. *J Urol* **158**: 12–22

Humphrey P, Wong A, Vogelstein B, Zalutsky M, Fuller G, Archer G, Friedman H, Kwatra M, Bigner S and Bigner D (1990) Anti-synthetic peptide antibody reacting at the fusion junction of deletion-mutant epidermal growth factor receptor in human glioblastoma. *Proc Natl Acad Sci USA* **87**: 4207–4211

Hunts J, Ueda M, Ozawa S, Abe O, Pastan I and Shimuzi N (1985) Hyperproduction and gene amplification of the epidermal growth factor receptor in squamous cell carcinomas. *Jpn J Cancer Res* **76**: 663–666

Kristt D and Yarden Y (1996) Differences between phosphotyrosine accumulation and Neu/Erb-2 receptor expression in astrocytic proliferative processes. *Cancer* **78**: 1272–1283

Laemmli U (1970) Cleavage of structural proteins during the assembly of head bacteriophage. *Nature* **227**: 680–685

Maddy S, Chisholm G, Hawkins R and Habib F (1987) Localisation of epidermal growth factor receptors in the human prostate by biochemical and immunocytochemical methods. *J Endocrinol* **113**: 147–153

Maddy S, Chisholm G, Busuttil A and Habib F (1989) Epidermal growth factor receptors in prostate cancer: correlation with histological differentiation of the tumour. *Br J Cancer* **60**: 41–44

Maygarden S, Strom S and Ware J (1992) Localisation of epidermal growth factor receptor by immunohistochemical methods in human prostatic carcinoma, prostatic intraepithelial neoplasia, and benign hyperplasia. *Arch Pathol Lab Med* **116**: 269–273

Mellon K, Thompson S, Charlton R, Marsh C, Robinson M, Lane D, Harris A, Horne C and Neal D (1992) p53, c-erbB-2 and the epidermal growth factor receptor in the benign and malignant prostate. *J Urol* **147**: 496–499

Modjtahedi H, Hickish T, Nicolson M, Moore J, Styles J, Eccles S, Jackson E, Salter J, Sloane J, Spencer L, Priest K, Smith I, Dean C and Gore M (1996) Phase 1 trial and tumour localisation of the anti-EGFR monoclonal antibody in head and neck or lung cancer. *Br J Cancer* **73**: 228–235

Morris G and Dodd J (1990) Epidermal growth receptor mRNA levels in human prostatic tumour cell lines. *J Urology* **143**: 1272–1274

Moscatello K, Holga-Madruga M, Godwin A, Ramirez G, Gunn G, Zoltick P, Biegel J, Hayes R and Wong A (1995) Frequent expression of a mutant epidermal growth factor receptor in multiple human tumours. *Cancer Res* **55**: 5536–5539

Moscatello D, Montgomery R, Sunderashan P, McDanel H, Wong M and Wong A (1996) Transformation and altered signal transduction by a naturally occurring mutant EGF receptor. *Oncogene* **13**: 85–96

Moscatello D, Ramirez G and Wong A (1997) A naturally occurring mutant human epidermal growth factor as a target for peptide vaccine immunotherapy of tumours. *Cancer Res* **57**: 1419–1424

Nagane M, Coufal F, Lin H, Bogler O, Cavenee W and Huang SH-J (1996) A common mutant epidermal growth factor receptor confers enhanced tumorigenicity on human glioblastoma cells by increasing proliferation and reducing apoptosis. *Cancer Res* **56**: 5079–5086

Newby J, Johnston S, Smith I and Dowsett M (1997) Expression of epidermal growth factor receptor and c-erbB2 during the development of tamoxifen resistance in human breast cancer. *Clin Cancer Res* **3**: 1643–1651

Nishakawa R, Ji X-D, Harmon R, Lazar C, Gill G, Cavenee W and Huang H-J (1994) A mutant epidermal growth factor receptor common in human gliomas confers enhanced tumorigencity. *Proc Natl Acad Sci USA* **91**: 7727–7731

Olapade-Olaopa E, MacKay E and Habib F (1998) Variability of immunohistochemical reactivity on stored paraffin slides (letter). *J Clin Pathol* **51**: 943

Olapade-Olaopa E, MacKay E, Taub N, Sandhu D, Terry T and Habib F (1999) Malignant transformation of human prostatic epithelium is associated with the loss of androgen receptor immuno-reactivity in surrounding stroma. *Clin Cancer Res* **5**: 569–576

Ozanne B, Richards C, Hendler F, Burns D and Gusterson B (1986) Overexpression of the EGF receptor is a hallmark of squamous cell carcinoma. *J Pathol* **149**: 9–14

Panneerselvam K, Kanakaj P, Raj S, Das M and Bishayee S (1995) Characterisation of a novel epidermal-growth-factor-related 200-kDa tyrosine kinase in tumour cells. *Eur J Biochem* **230**: 951–957

Putz T, Culig Z, Eder I, Nessler-Menardi C, Bartsch G, Grunike H, Uberall F and Klocker H (1999) Epidermal growth factor (EGF) receptor blockade inhibits the action of EGF, insulin-like growth factor I, and a protein kinase A activator on the mitogen-activated protein kinase pathway in prostate cancer cell lines. *Cancer Res* **59**: 227–233

Ro J, North S, Gallick G, Hortobagyi G, Gutterman J and Blick M (1988) Amplified and overexpressed epidermal growth factor receptor gene in uncultured primary human breast carcinoma. *Cancer Res* **48**: 161–164

Sainsbury J, Farndon J, Needham G, Malcom A and Harris A (1987) Epidermal growth factor receptor status as predictor of early recurrence and death from breast cancer. *Lancet* **1**: 1398–1402

Sakr W and Grigon D (1997) Prostate cancer: indicators of aggressiveness. *Eur Urol* **32**: 15–23

Schmidt M, Reisser P, Hills D, Gullick W and Wels W (1998) Expression of an oncogenic mutant EGF receptor markedly increases the sensitivity of cells to an EGF-receptor-specific antibody-toxin. *Int J Cancer* **75**: 878–884

Sherwood E, Dongen JV, Wood C, Liao S, Kozlowski J and Lee C (1998) Epidermal growth factor receptor activation in androgen-independent but not androgen-stimulated growth of human prostatic carcinoma cells. *Br J Cancer* **77**: 855–861

Thompson D and Gill G (1985) The EGF receptor: structure, regulation and potential role in malignancy. *Cancer Surv* **4**: 767–788

Tukeri L, Sakr W, Wykes S, Grigon D, Pontes J and Macoska J (1994) Comparative analysis of epidermal growth factor receptor gene expression and protein product in benign and malignant prostate tissue. *Prostate* **25**: 199–205

Ulrich A, Coussens L, Hayflick J, Dull T, Gray A, Tam A, Lee J, Yarden Y, Libermann T, Schlessinger J, Downward J, Maves E, Whittle N, Waterfield M and Seeberg P (1984) Human epidermal growth factor receptor cDNA sequence and aberrant expression of the amplified gene in A431 epidermoid carcinoma cells. *Nature (London)* **309**: 418–425

Wikstrand C, Hale L, Batra S, Hill M, Humphrey P, Kurpad S, McLendon R, Moscatello D, Pegram C, Reist C, Traweek S, Wong A, Zalutsky M and Bigner D (1995) Monoclonal antibodies against EGFRvIII are tumor specific and react with breast and lung carcinomas and malignant gliomas. *Cancer Res* **55**: 3140–3148

Wong A, Bigner S, Bigner D, Kinzler K, Hamilton S and Vogelstein B (1987) Increased expression of the EGF receptor gene in malignant gliomas is invariably associated with gene amplification. *Proc Natl Acad Sci USA* **84**: 6899–6903

Wong A, Ruppert J, Bigner S, Grzechick C, Humphrey P, Bigner D and Volgstein B (1990) Structural alterations of the epidermal growth factor receptor gene in human gliomas. *Proc Natl Acad Sci USA* **87**: 4207–4211

Wong A, Ruppert J, Bigner S, Grzeschik C, Humphrey P, Bigner D and Vogelstein B (1992) Structural alterations of the epidermal growth factor receptor gene in human gliomas. *Proc Natl Acad Sci USA* **89**: 2965–2969

Xu Y, Richert N, Ito S, Merlino G and Pastan I (1984) Characterisation of epidermal growth factor receptor gene expression in malignant and normal human cell lines. *Proc Natl Acad Sci USA*, **81**: 7308–7312

 © 2000 Cancer Research Campaign
